# 色谱在糖组学分析中的应用

**DOI:** 10.3724/SP.J.1123.2023.12003

**Published:** 2024-07-08

**Authors:** Yi ZHENG, Cuiyan CAO, Zhimou GUO, Jingyu YAN, Xinmiao LIANG

**Affiliations:** 1.中国科学院大连化学物理研究所,辽宁 大连 116023; 1. Dalian Institute of Chemical Physics, Chinese Academy of Sciences, Dalian 116023, China; 2.赣江中药创新中心,江西 南昌 330100; 2. Ganjiang Chinese Medicine Innovation Center, Nanchang 330100, China

**Keywords:** 液相色谱, 糖组学, 糖链富集, 糖链分离, liquid chromatography (LC), glycomics, glycan enrichment, glycan separation

## Abstract

糖组学是继基因组学和蛋白组学后发展起来的组学技术,是研究细胞、组织或生物体内糖组的组成、结构及功能的一门学科。糖组学研究对深入了解生命活动规律、疾病的预防和治疗以及药物的质控和研发具有重要意义。糖组在生物样品中的丰度较低,且受单糖组成、糖苷键连接位置、连接方式、分支结构等因素的影响,使得糖链的组成与结构复杂多样,给糖组学研究带来了巨大挑战。液相色谱分离技术以及液相色谱-质谱联用技术被广泛应用于糖组的结构解析,在糖组学研究中发挥了不可或缺的作用。本文首先从色谱分离原理的角度讨论了糖链的富集方法及其在糖组学研究中的应用案例,并比较分析了各种方法的优势与不足;在糖链的分离与分析方面,从色谱分离模式的角度,分别列举了反相色谱、高效阴离子色谱、亲水相互作用色谱和多孔石墨化碳色谱分离糖链、糖缀合物或糖衍生物的分离原理,以及各类色谱模式联合质谱解析在糖组学分析中的研究进展。本文对各类色谱方法的分离特色进行了总结与讨论,为研究人员针对特定样品或特定目标物的分离与分析选择合适的方法提供了参考。近年来,在色谱技术的推动下,糖组学研究取得了相当大的进展。随着色谱新材料和新方法的不断开发,色谱技术将在糖组学研究中发挥更加重要的作用。

## Abstract (en)

Glycomics, an emerging “omics” technology that was developed after genomics and proteomics, is a discipline that studies the composition, structure, and functions of glycomes in cells, tissues, and organisms. Glycomics plays key roles in understanding the laws of major life activities, disease prevention and treatment, and drug quality control and development. At present, the structural analysis of glycans relies mainly on mass spectrometry. However, glycans have low abundance in biological samples. In addition, factors such as variable monosaccharide compositions, differences in glycosidic bond positions and modes, diverse branching structures, contribute to the complexity of the compositions and structures of glycans, posing great challenges to glycomics research. Liquid chromatography can effectively remove matrix interferences and enhance glycan separation to improve the mass spectrometric response of glycans. Thus, liquid chromatography and liquid chromatography coupled with mass spectrometry are important technical tools that have been actively applied to solve these problems; these technologies play indispensable roles in glycomics research. Different studies have highlighted similarities and differences in the applications of various types of liquid chromatography, which also reflects the versatility and flexibility of this technology.

In this review, we first discuss the enrichment methods for glycans and their applications in glycomics research from the perspective of chromatographic separation mechanisms. We then compare the advantages and disadvantages of these methods. Some glycan-enrichment modes include affinity, hydrophilic interactions, size exclusion, and porous graphitized carbon adsorption. A number of newly developed materials exhibit excellent glycan-enrichment ability. We enumerate the separation mechanisms of reversed-phase high performance liquid chromatography (RP-HPLC), high performance anion-exchange chromatography (HPAEC), hydrophilic interaction chromatography (HILIC), and porous graphitic carbon (PGC) chromatography in the separation and analysis of glycans, and describe the applications of these methods in the separation of glycans, glycoconjugates, and glyco-derivatives. Among these methods, HILIC and PGC chromatography are the most widely used, whereas HPAEC and RP-HPLC are less commonly used. The HILIC and RP-HPLC modes are often used for the separation of derived glycans. The ionization efficiency and detectability of glycans are significantly improved after derivatization. However, the derivatization process is relatively cumbersome, and byproducts inevitably affect the accuracy and completeness of the detection results. HPAEC and PGC chromatography exhibit good separation effects on nonderivative glycans, but issues related to the detection integrity of low-abundance glycans owing to their poor detection effect continue to persist. Therefore, the appropriate analytical method for a specific sample or target analyte or mutual verification must be selected.

Finally, we highlight the research progress in various chromatographic methods coupled with mass spectrometry for glycomics analysis. Significant progress has been made in glycomics research in recent years owing to advancements in the development of chromatographic separation techniques. However, several significant challenges remain. As the development of novel separation materials and methods continues, chromatographic techniques may be expected to play a critical role in future glycomics research.

生物体或细胞在特定条件下产生的一整套聚糖和糖缀合物被称为糖组^[1]^。与蛋白质组相似,每种细胞类型都有自己独特的糖组,由基因和代谢状态共同调控表达。哺乳动物的糖组主要包括与糖蛋白、糖脂、蛋白聚糖和糖基磷脂酰肌醇等糖缀合物共价连接的糖链、糖胺聚糖和游离的低聚糖等^[1]^。近年来,糖组被发现与众多的生理、病理过程密切相关,例如细胞表面的糖缀合物糖链调节多种受体-配体相互作用,是许多微生物的靶点^[2]^,参与调节细胞黏附和发育,影响癌症细胞的增殖和转移^[3]^等;分泌途径中的聚糖参与调节蛋白质的质量控制、转运和分泌^[4]^;细胞核质糖基化水平的调节对细胞功能有着深远的影响^[5]^;蛋白糖基化的异常可以导致疾病的发生,目前已发现了160余种与先天性糖基化障碍相关的疾病^[6]^。研究表明,生物体内源性蛋白的异常糖基化水平也会随着生活方式和病理条件发生不同程度的改变,如癌症、慢性炎症性疾病、感染性疾病、自身免疫性疾病等^[7]^。此外,糖基化被认为是生物治疗药物生产中的一个关键质量指标,糖链会极大地影响蛋白药物的安全性和有效性等。因此,糖组的研究对深入了解生命活动规律以及疾病的预防和治疗以及药物质控和开发具有重要意义。

糖组学是继基因组学和蛋白组学后发展起来的组学技术,是研究细胞、组织或生物体内糖组的组成、结构及其功能的一门学科^[8]^。与基因和蛋白质相比,糖的生物合成是一个非模板驱动过程,受到多种因素的影响^[9]^,如糖基转移酶的表达和细胞环境等。因此,糖组的组成和结构更为复杂,主要表现在以下几个方面:(1)单糖种类多,脊柱动物体内主要有9种单糖,不同单糖组成、数目和序列的变化都会产生不同的糖链结构;(2)糖苷键连接位置多变,单糖是多羟基醛(酮)的结构,尽管在生物体中并非每种单糖的所有羟基都可作为糖苷键的位点,但多个羟基连接位点急剧增加了糖链结构的可能性;(3)糖苷键连接方式改变,糖链结构中每个糖苷键都可能存在α和β两种连接方式;(4)不同于蛋白质和核酸的线性结构,糖链可形成分支结构,分支结构的发生位点、数目、序列组成和长短又会增加糖链的复杂性;(5)同分异构体多,糖链结构在上述几个特点的组合下形成了大量丰富的同分异构体,也成为糖组复杂性的最重要特征^[10]^。因此,糖组的组成和结构是糖组学研究中最主要的难点之一。目前,糖链的结构解析主要依赖于质谱技术^[11]^。但受限于糖链在生物样品中的丰度低、含量差异大,且糖分子不易被电离等因素,糖链的精细结构解析仍存在巨大挑战。液相色谱(LC)分离技术为去除基质干扰、提高糖链丰度、改善质谱响应以及异构体分离提供了良好的解决方案。如图1所示,糖组的组成和结构研究大致流程为特定生物样品中糖链的释放、糖链的富集、糖链的分离以及糖链的结构解析和定量^[12]^。

**图1:**
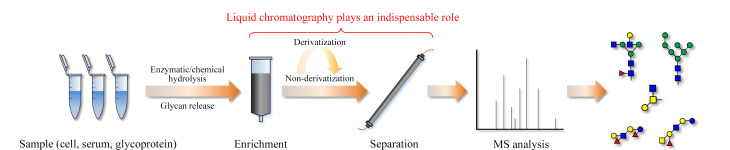
糖组学的研究流程示意图以及色谱在其中发挥作用的关键步骤

在整个分析过程中,各类LC技术参与了比较关键的糖链富集和分离过程,对于提高糖链的丰度与纯度以及增加糖链的检测响应发挥了不可或缺的作用。在不同的研究报道中,各类LC的应用场景和应用对象既有相似之处,也存在差异,体现了色谱技术在糖组学研究中的普适性和灵活性。为此,本文从色谱分离模式的角度,介绍、评述了文献报道的各类LC技术在糖组学研究中的应用,并对其发展前景进行了展望。

## 1 液相色谱应用于糖链的富集

在生物体中,除了游离的低聚糖外,其他类型的糖链都以糖缀合物的形式与其他分子共价连接。由于糖链的极性大、丰度低,在质谱检测时,受其他基质干扰严重,且缺少易带电基团而不易被检测。因此,在结构分析前需对糖链进行预分离,即对聚糖或糖缀合物上的糖链进行释放和“捕获”富集。糖链的释放方法很多,例如,糖蛋白上N-糖链可以利用高特异性的糖苷酶(如PNGase F^[13]^、PNGase A^[14]^和Endo H^[15]^等)或非特异性的蛋白水解酶(如pronase E结合glycosylasparaginase^[16]^)酶解释放。O-糖采用化学法来释放,包括*β*-消除法^[17]^和氧化降解法^[18]^等。此外,为了增加糖链的带电情况、降低水解难度,大量研究还采用将糖蛋白降解为糖肽的策略,来分析糖肽上的糖链结构。

糖链被释放后,需要通过富集来提高丰度,便于后续的组成和结构分析。糖链的富集主要依赖于糖类多羟基结构、亲水性强等特点,可分为共价富集法(基于硼酸或酰肼的富集法、衍生标签辅助富集法等^[19]^)和非共价富集法。其中,非共价富集法根据操作过程和应用场景的不同还可分为固相萃取法(SPE)和LC富集法。其中,SPE是由色谱理论发展的一种固-液相萃取的物理过程,本质上也是一种LC。其结合和洗脱过程与LC相似,主要包括填料的装填和活化、上样、淋洗(去除干扰物)以及洗脱(用小体积的溶剂将目标物洗脱)收集。SPE主要为手动或半自动操作,操作简单、快速,无需特殊装置,可小型化或微量化^[20]^。因此,SPE是糖组学研究中富集糖链或糖肽最主要的方法。而LC富集法有别于SPE,富集材料常被装填于高效液相色谱(HPLC)柱中,通过HPLC系统,可实现对糖链或糖肽的自动化快速富集。本文将从分离模式的角度对糖链的富集方法进行分别介绍。

### 1.1 亲和作用模式

亲和色谱(affinity chromatography)是利用生物系统中存在的特异性和可逆性的相互作用进行分离的色谱技术,该方法特异性强,选择性高。一些特定结构的糖分子(如凝集素等)可以与某些生物分子可逆地结合,因此,亲和色谱也是富集聚糖和糖肽常用的方法之一。例如,Llop等利用接骨木凝集素琼脂糖凝胶微型色谱柱富集、分析了不同侵袭性前列腺增生^[21]^和前列腺癌患者^[22]^血清样本中的唾液酸化N-聚糖;Yue等^[23]^在Ti-IMAC(Ⅳ)材料(immobilized metal ion affinity chromatography with Ti^4+^)的基础上建立了一种多重作用方式的富集方法,该方法利用2 mg Ti-IMAC(Ⅳ)材料装填微型的SPE柱,通过亲和相互作用联合亲水相互作用,对人血清中完整的O-GalNAc糖肽进行了富集;Ruiz-May等^[24]^系统总结并详细介绍了不同凝集素亲和色谱富集不同类型N-糖链的操作与使用方法等。然而,亲和色谱识别的高度特异性导致其很难富集所有结构类型的糖链。因此,该方法在富集聚糖方面的应用较为有限。

### 1.2 亲水相互作用模式

亲水色谱材料也常被用于糖链或糖肽的富集。例如,键合有两性离子的商品化ZIC^®^-HILIC SPE柱常被用于糖蛋白糖链或糖肽的富集^[25,26]^; Selman等^[27]^利用棉絮中纤维素的多羟基结构,发展了棉絮SPE方法对人免疫球蛋白G(immunoglobulin G, IgG)的糖链和糖肽分别进行富集;我们利用课题组研发的键合天冬氨酸的亲水材料(Click AA)装填的SPE柱成功对人IgG水解产物中的糖肽进行富集^[28]^;此外,我们还利用键合组氨酸的亲水材料(HBS, histidine bonded silica)填充的微量SPE柱对人血清中糖肽进行了富集,从2 mL的人血清中鉴定出487个糖基化位点,富集选择性达到92%^[29]^。有别于亲水作用SPE,亲水相互作用色谱(hydrophilic interaction chromatography, HILIC)通常是一种在线富集模式,即一般以较短的捕集柱形式作为液相色谱系统的一部分,可以更加快速、高效地对目标糖链或糖肽进行富集。TSKgel Amide-80是一种经典的HILIC材料,在被Yoshida^[30]^用来分离肽段后常被用于糖链或糖肽的分离与富集。例如,Ruhaak等^[31]^利用两根TSKgel-Amide 80捕获柱(10 mm×2.0 mm)在线对人血清中总糖肽进行了纯化富集;Lam等^[32]^利用微升级液相系统Amide-80亲水捕获柱(20 mm×150 μm)对不同蛋白水解产物中的N-糖肽进行富集,经后续结构分析,发现了火鸡卵清蛋白中的23种新型聚糖结构。此外,其他类型的亲水色谱柱也被用于糖链和糖肽的富集,例如,Shu等^[33]^利用Ultimate HILIC amphion色谱柱(100 mm×4.6 mm)对人血清中完整的糖肽进行了富集,便于后续的酶解及糖链结构分析;本课题组^[34]^利用键合了谷胱甘肽的Click TE-GSH亲水色谱柱(50 mm×2.1 mm)在线对母乳寡糖混合物中的中性糖进行了富集,巧妙地利用Click TE-GSH的静电排斥作用将酸性糖纯化,进行下一维色谱分析;Liu等^[35]^还利用键合了麦芽糖的Click Maltose亲水色谱柱(50 mm×2.1 mm)在超高效液相色谱上建立了在线糖肽富集方法,对健康人和前列腺癌患者血清中的糖链进行富集与比较分析。

### 1.3 尺寸排阻模式

与蛋白质相比,释放下来的糖链相对分子质量小得多。因此,利用尺寸排阻色谱(size exclusion chromatography, SEC),糖链可以很容易地从蛋白质的混合物中分离出来。例如,Qin等开发了一种氧化介孔碳材料O-CMK-3^[36]^和一种形状均匀、介孔结构高度有序的硅碳复合纳米颗粒(NP-MCM-C)^[37]^,利用介孔对蛋白质的排阻效应以及碳和聚糖之间的相互作用,有效富集了血清等生物样品中的N-糖链;Sun等^[38]^设计合成了石墨烯/介孔二氧化硅复合材料(C-graphene@mSiO_2_),该材料具有大表面积和均匀的孔径,同样利用尺寸排阻能力和石墨化碳与聚糖之间的相互作用,从卵清蛋白消化液和400 nL正常人血清中分别富集得到25种和48种N-糖链。

### 1.4 多孔石墨化碳(porous graphitic carbon, PGC)富集模式

PGC对于极性分子,特别是对糖类具有显著的选择性,也被广泛应用于糖类化合物的富集。例如,Hahn等^[39]^利用PGC-SPE对不同母乳样品中的低聚寡糖进行了分别富集,后续比较分析了不同母乳中寡糖的差异;Seo等^[40]^利用PGC-SPE富集与液相色谱-质谱(LC-MS)联用技术分析了从溶酶体酶中释放的天然唾液酸化聚糖;Kolarich等^[41]^利用PGC-SPE对猪血浆中总糖蛋白的N-糖链进行了富集等。

### 1.5 其他富集材料与方法

“糖捕获”技术离不开材料学的发展。近年来,为了满足微量样品分析的要求,越来越多的新型材料和新方法被不断开发。金属有机骨架(metal organic frameworks, MOFs)是一类由金属和有机连接臂自组装形成的多孔材料,被证明是满足糖肽富集需求的优秀候选材料^[42]^。例如,He等^[43]^报道了一种亲水的中空锆有机框架材料(HHZr-MOFs),从10 μL口腔炎症患者的唾液样本中同时捕获了98个内源性磷酸肽和216个内源性N-糖肽;Zhou等^[44]^报道的二维中空硫化钴纳米叶片材料(ZIF-L-Co-S-Au-Cys)从IgG标准样品的酶解液和人血浆样品中富集了N-糖肽。新型聚合物材料也被报道用于微量样品中糖肽的富集。例如,聚((*N*-异丙基丙烯酰胺-co-4-(3-丙烯酰硫基)苯甲酸0.2)材料(PNI-co-ATBA0.2)实现了从50 μg HeLa细胞裂解液中同时富集631个磷酸化肽和120个糖肽^[45]^; Wang等^[46]^报道了多组氨酸修饰微球,通过静电作用、亲水相互作用等协同作用,从复杂的生物样品中富集唾液化糖肽。磁性固相萃取材料具有制备简单、成本低、磁响应好等优点,也被用于体液中糖蛋白和糖肽的定量分析^[47]^; Huan等^[48]^报道的磁性纳米纤维材料magHN/Au GSH对糖肽富集具有令人满意的特异性,仅从1 μL人血清中就鉴定出246个N-糖肽;本课题组利用乳液界面聚合制备的聚丙烯酰胺-聚苯乙烯/聚二乙烯基苯系列亲水-疏水异质结构纳米孔微球实现了从复杂生物流体中富集低丰度的糖肽^[49]^; Xiong等^[50]^对近年来发展的糖肽富集新材料与新方法进行了系统的总结。此外,Aguedo等^[51]^报道的类石墨烯结构的MXene纳米材料实现了N-糖链的富集;Sha等^[52]^利用纤维素微球实现了人和动物糖蛋白糖链和糖肽的富集;通常在蛋白质组学研究中用于捕获磷酸化肽的金属氧化物SPE材料如二氧化钛(TiO_2_)和氧化锆(ZrO_2_)也被报道用于唾液酸化糖链的富集^[53]^。

表1列举了上述部分富集方法及其优缺点,研究者需要根据所分析的目标物特点进行合理选择,还可将多种方法联合使用,以达到最佳的富集效果。

**表1:** 不同的糖链或糖肽富集方法比较

Enrichment method	Stationary phase or column	Applications	Eluent	Advantages and limitations	Ref.
AFC	*Sambucus nigra* agglutinin agarose	core fucosylation and the sialic acid linkage of prostate-specific anti- gens, N-glycans of serum samples	0.5 mol/L lactose in 1%BSA	good selectivity and high efficiency, but lack of general applicability	[21]
HILIC-SPE	SeQuant ZIC-HILIC	glycopeptides from human serum	1% TFA aqueous solution	universality, but	[25]
	cotton wool pads	IgG Fc N-glycans and N-glycopeptides	water	unsatisfactory repeatability	[27]
	histidine-bonded silica	N-glycopeptides from BSA/fetuin mixture digests, human serum tryptic digests	40% ACN/5 mmol/L NH_4_HCO_3_ (pH 8.2)		[29]
HILIC	TSKgel Amide 80	2-AA-labeled N-glycans of human plasma	80% ACN, 20% 50 mmol/L ammonium formate (pH 4.4)	universality and rapidity, but low selectivity owing	[31]
	Ultimate HILIC amphion column	N-glycopeptides of horseradish per- oxidase, ovalbumin, human holo- transferrin, and human serum	A: 0.1%TFA; B: 0.1%TFA in ACN; 0-20 min, 80%B; 21-30 min, 2%B; 31-38 min, 80%B	to nonspecific adsorption	[33]
	Click Maltose	N-glycopeptides of IgG, BSA and human serum with and without pancreatic tumors	A: 0.1%TFA; B: 0.1%TFA/98% ACN; 0-11 min, 82%B; 11-13 min, 30%B; 13-15 min, 0B		[35]
SEC	oxidized meso- porous carbon ma- terial (O-CMK-3)	N-glycans of hen ovalbumin, BSA, human serum from healthy volun- teers and patients with liver cancer	50% ACN solution	rarely applied, SEC only used as an auxiliary of carbon materials	[36]
PGC-SPE	PGC cartridges	human milk oligosaccharides	20% ACN in water and 40% ACN in 0.05%TFA aqueous solution	universality and high enrichment scale, but	[39]
	PGC cartridges	N-glycans of recombinant human *α*-galactosidase	steps: 1) 20% ACN in H_2_O; 2) 10% ACN in H_2_O with 0.05% TFA; 3) 40% ACN in H_2_O with 0.05%TFA	unsatisfactory stability and repeatability	[40]
Metal oxides- SPE	combined TiO_2_/ZrO_2_ resin	N-glycans with mono- or diphospho-esters of recombinant *β*-glucuronidase	digested with calf intestinal alkaline phosphatase on resin in 50 mmol/L Tris-HCl, pH 9.3, 1 mmol/L MgCl_2_, 0.1 mmol/L ZnCl_2_, and 1 mmol/L spermidine at 37 ℃ overnight	lack of general applicability	[53]

AFC: affinity chromatography; HILIC: hydrophilic interaction chromatography; SEC: size exclusion chromatography; PGC: porous graphitic carbon; IgG: immunoglobulin G; BSA: bovine serum albumin; TFA: trifluoroacetic acid; ACN: acetonitrile; 2-AA: 2-aminobenzoic acid.

## 2 液相色谱应用于糖链的分离分析

LC-MS是结构糖组学研究中主要的技术手段。MS是糖结构解析的核心,而LC不但是MS解析准确性的保障,还提供了用于糖结构分析、定量的保留时间和峰面积等关键信息。在糖组学分析中,用于糖链分离的色谱方法很多,根据分离模式可分为反相高效液相色谱法(RP-HPLC)、高效阴离子交换色谱法、HILIC和多孔石墨化碳色谱法。

### 2.1 反相高效液相色谱分离

糖链亲水性强,没有发色基团,无法以常规的反相色谱分离和紫外-可见光或荧光检测器进行高灵敏度检测。因此,解决策略之一是释放糖链,保留疏水基团或发色基团,如将糖蛋白酶解为糖肽进行分析。例如,Falck等^[54]^利用胰蛋白酶酶解IgG制备了糖肽,使用纳升级Ascentis Express C18 nanoLC反相色谱柱(50 mm×75 μm)分离,联合MS解析,建立了一种高通量的糖组分析方法。由于糖肽在反相色谱上的分离是由糖肽的肽段部分决定,而不是由糖链部分决定,因此不同IgG亚型形成3个不同保留时间的糖肽簇。糖链在反相色谱上分析的另一个解决策略是衍生化,通过在聚糖和糖肽的结构上化学键合一个额外的化合物“标签”,来增加分析物的色谱保留机制,并改善聚糖类的低电离效率和可检测性。例如Lattová等^[55]^利用苯肼衍生化释放的N-糖链,结合Vydac 218 TP54 C18柱(250 mm×4.6 mm)分离与MALDI-MS解析,分析了患有头颈癌小鼠血清中的N-糖组;Wang等^[56]^发展了高效的“一锅法”分析,采用1-苯基-3-甲基-5-吡唑啉酮(PMP)衍生糖链联合反相色谱(SinoChrom ODS-BP色谱柱,250 mm×4.6 mm)分离与MS联用,同时解析了人精浆中完整的N-糖和O-糖,如图2所示;Braun等^[57]^建立了邻苯二醛和3-巯基丙酸标记、Luna Omega C18反相色谱柱(150 mm×4.6 mm)分离结合荧光检测器分析糖胺聚糖的方法,比较分析了不同物种来源的胶原中糖胺聚糖的差异。此外,柱前衍生化联合RP-HPLC还常被用于植物多糖^[58⇓-60]^、细菌胞外多糖^[61,62]^、真菌多糖^[63⇓-65]^等多糖样品的单糖组成分析。但对于带有亲脂性基团的糖缀合物可以采用RP-HPLC直接进行分析,例如对神经节苷脂^[66,67]^和细菌脂多糖^[68]^的分离分析等。

**图2:**
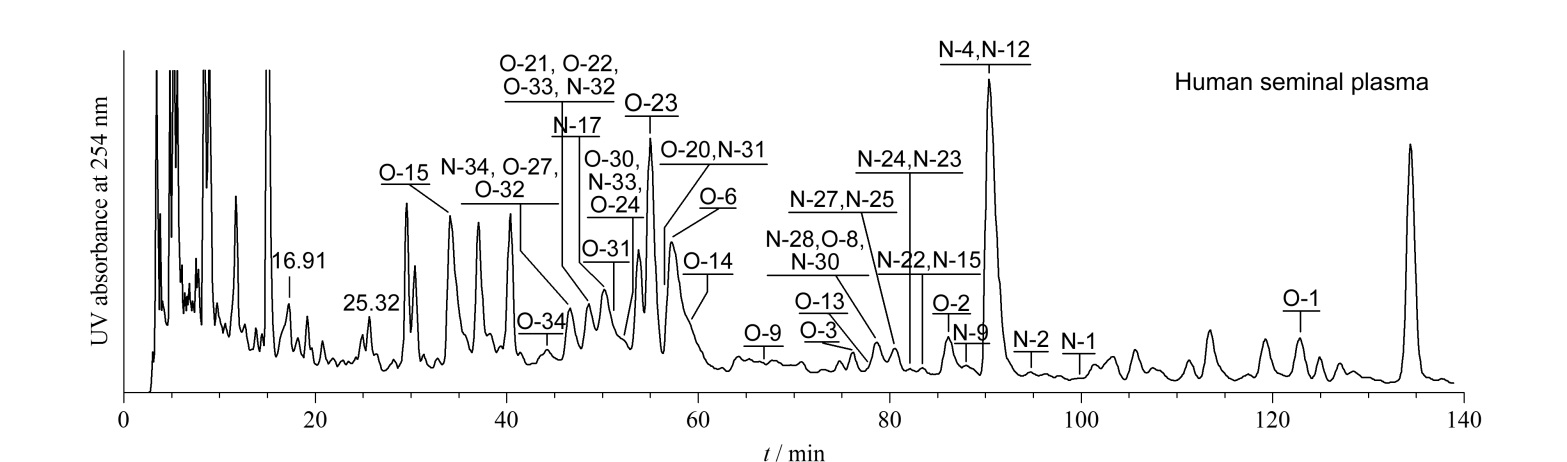
精浆中O-糖和N-糖的RP-HPLC-MS/MS在线分析色谱图

### 2.2 高效阴离子交换色谱分离

高效阴离子交换色谱(HPAEC)与脉冲安培检测器/脉冲电化学检测器(PAD/PED)相结合是测定糖类最常用的技术之一。糖分子中的羟基可电离,显示弱酸性。以单糖为例,葡萄糖具有几个潜在的可电离羟基,其酸度等级如下:1-OH>2-OH≥6-OH>3-OH>4-OH^[69]^。许多糖类化合物的p*K*_a_值在12~14范围内^[70]^。因此,在高pH条件下,聚糖的羟基部分或全部转化为含氧阴离子,从而能够在阴离子色谱柱上被选择性分离。糖类分离最常用的HPAEC柱是Dionex CarboPac系列(Thermo Fisher Scientific,美国)色谱柱,其中CarboPacPA100和CarboPacPA 200被经常用于聚糖的分离和结构表征。HPAEC中使用氢氧化钠的水溶液作为流动相来保持高pH条件,而为了洗脱聚糖,通常需要在氢氧化钠溶液中加入一定浓度的乙酸钠。PAD常与HPAEC联用,糖类的检测通常在金、铂等固体电极上采用四电位波形进行^[71]^。

HPAEC-PAD被广泛用于低聚糖的分析,例如植物来源的低聚糖^[72⇓-74]^、真菌低聚糖^[75,76]^、低相对分子质量肝素^[77]^、乳寡糖^[78]^以及N-糖^[79]^等。通常,MS被认为与HPAEC中使用的高碱性洗脱剂不相容。然而,在线脱盐抑制器(Thermo Fisher Scientific,美国)的问世将HPAEC与MS兼容,极大地促进了HPAEC在聚糖结构解析中的应用。例如,Maier等^[80]^建立了mini-bore HPAEC-MS/MS分析方法,采用CarboPac PA200微孔柱(250 mm×1 mm)分离,经过在线脱盐串联MS的方式,对人血清中IgG和单克隆抗体IgG的Fc段N-糖组进行了比较分析;Szabo等^[81]^同样采用CarboPac PA200(250 mm×3 mm)柱,经过在线脱盐串联MS的方式,分析了3种模型糖蛋白的N-糖组,共鉴定了125种N-糖链,并发现了17种新型N-糖结构。其结果显示,HPAEC对唾液酸化的N-糖分离效果较好;此外,Szabo等^[82]^还利用人红细胞生成素蛋白比较了HPAEC-MS模式与2-氨基苯甲酰胺(2-aminobenzamide, 2-AB)衍生化结合HILIC-MS模式分析N-糖组的差异性,发现两种模式有各自的优势和特点:HPAEC-MS保留了完整的唾液酸化、磷酸化和硫酸化的N-糖链,但没有显示唾液酸O-乙酰化;而2-AB衍生化-HILIC-MS模式表现出对唾液酸乙酰化的较好回收率,但唾液酸化聚糖定量的准确性存在一定问题;因此该文献作者认为采用两种模式互补分析可以反映完整N-糖组的实际情况。

### 2.3 亲水相互作用色谱分离

HILIC是糖组学分析中最常用的分离技术之一。HILIC可认为是一种正相色谱,通常使用极性化合物键合固定相,使用水溶性有机溶剂(ACN、丙酮或甲醇)-水作为流动相进行结合和洗脱。HILIC的分离机制比较复杂。一般条件下,流动相中的水会在固定相的极性表面形成一个富水层,亲水性分析物优先进入这种富水层形成相互作用(亲水分配作用、氢键作用和静电相互作用)从而被保留,而低亲水性的分析物被分配在高有机组分的流动相中^[83]^。但在特定条件下,如采用高有机组分(30%~70%)的流动相洗脱时,亲脂性的分析物也会与疏水的硅氧烷基团形成反相保留作用或形成离子交换相互作用^[84,85]^。通常,分析物在HILIC上的保留取决于其亲水电位。对于非衍生的聚糖,亲水电位受到糖分子极性基团数量、分子尺寸、电荷和分支等因素影响。糖链的主要亲水基团是羟基、乙酰氨基和羧基。糖链上亲水基团的数量通常与糖的聚合度(degree of polymerization, DP)呈正相关,因此HILIC的分离通常显示保留与DP之间的相关性。然而,HILIC对结构相似的糖链异构体分离能力并不理想。在糖组学分析中,科学家们也常采用HILIC模式分离衍生化的糖链,如最常用的2-AB衍生化糖链^[11,86]^和PMP衍生化糖链^[87,88]^等。

HILIC的固定相有许多种类型。传统的HILIC固定相一般是硅胶。随着HILIC技术的发展,在糖的分离研究中出现了不同类型键合配体的固定相,如醇基键合、氨基键合、酰胺键合、聚合物键合和两性离子键合等。例如,Yang等^[89]^利用五醇基键合的Halo 90 Å penta-HILIC柱(150 mm×2.1 mm)偶联MS对奶牛和牦牛乳中糖蛋白的非衍生N-糖组进行了比较分析,分离鉴定了80种N-糖链;Radovani等^[90]^利用酰胺键合的BEH amide柱(100 mm×2.1 mm)串联MS,建立了一种高效的N-糖组分析方法,比较分析了唾液和血浆中IgG的N-糖谱,评估了唾液IgG的N-糖链在不同储存条件下的稳定性;Staples等^[91]^利用Amide-80固定相装填微型色谱柱(150 mm×75 μm)串联MS建立了糖胺聚糖组分析方法分析不同结构的肝素;Wu等^[92]^利用Accucore 150 Amide HILIC色谱柱(250 mm×2.1 mm)结合在线和离线MS/MS建立了分析肝素结构的方法等。我们课题组也发展了一系列亲水性固定相,用于分离和纯化极性化合物。其中,键合天冬氨酸的“ABS”材料被用于人乳寡糖组的分离分析^[93]^(见图3),通过“炔基-叠氮化物(alkyne-azide)”点击化学合成的麦芽糖基键合HILIC材料“Click Maltose”被用于卵清蛋白N-糖肽的分离^[94]^等。

**图3:**
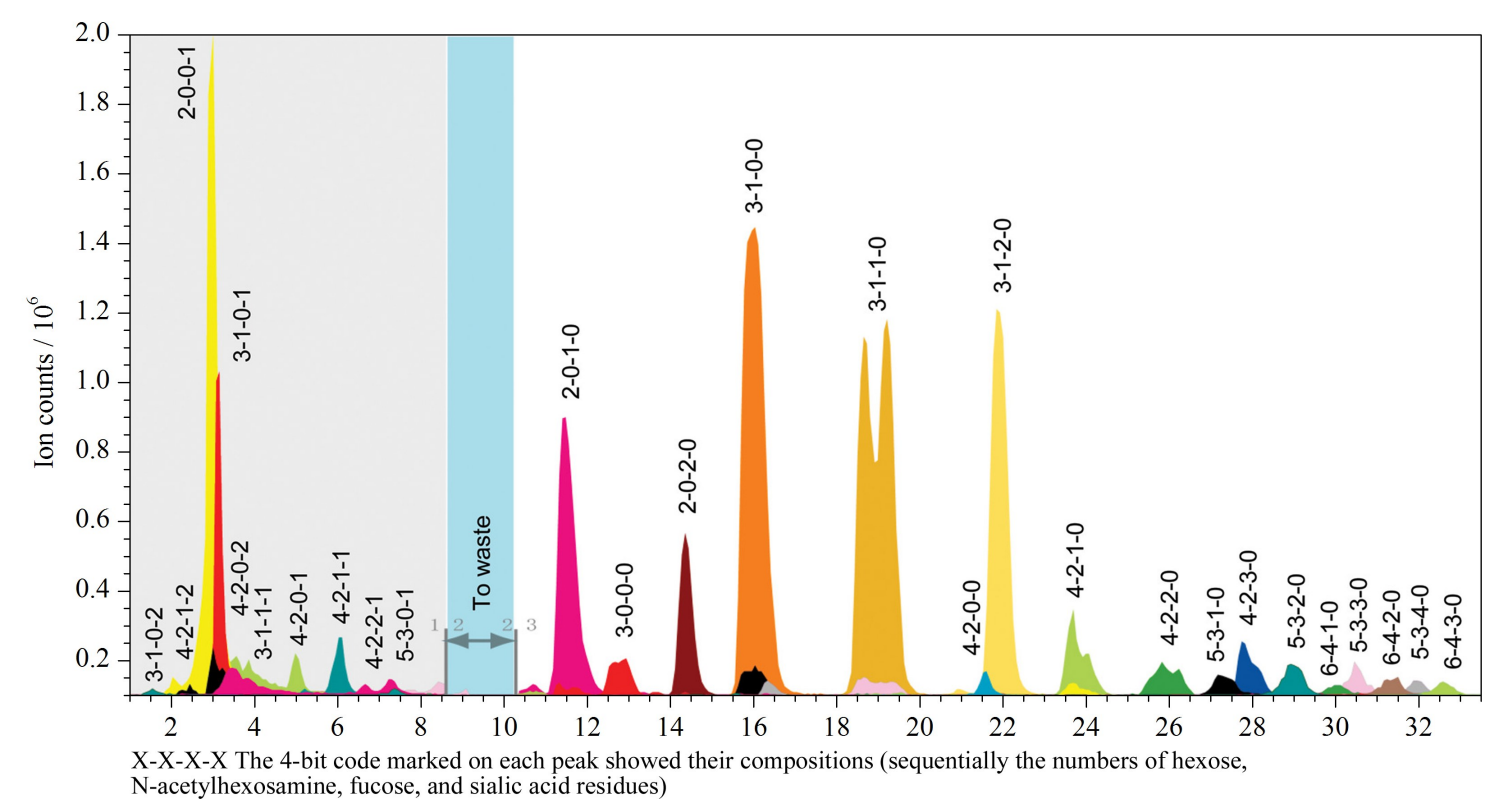
人乳寡糖的HILIC-MS/MS分析

### 2.4 多孔石墨化碳色谱分离

PGC自20世纪80年代首次作为LC固定相以来^[95]^,已被应用了40余年。它被证明可用于极性分子的分离,特别是对糖类具有显著的选择性。PGC的石墨片由*sp*^2^杂化的碳原子组成,呈六边形排列,碳原子通过共价键连接。石墨薄片通过范德华相互作用而交织在一起,赋予其高刚性和机械稳定性^[95]^。虽然PGC经常被用于糖类化合物的分离,但糖类在PGC上的保留机制尚不完全清楚,一般认为是疏水相互作用和极性保留效应^[96]^。PGC在分离糖类方面具有超高的分辨能力和良好的分离选择性,不仅可用于糖链异构体的分离,甚至可以将还原性糖的差向异构体分离^[96⇓-98]^,即将同一化合物还原端分别为*α*-和*β*-构象的异构体分成一对色谱峰。为了降低糖链差向异构体分离导致的色谱图复杂问题,研究者通常在色谱分离分析前,将还原性寡糖的醛基采用氰基硼氢化钠还原成醇基或衍生化试剂(如2-AB、PMP等)处理^[11,56,86⇓-88,99]^。虽然该法大大提高了糖链的检测灵敏度,改善了糖链的色谱分离效果,但还原端的醛基被还原成醇基后,不利于后续的原位串联质谱结构分析与基于糖芯片的糖组学功能研究^[100,101]^。

根据研究报道,具有不同结构的各种非衍生的低聚糖,如母乳低聚糖^[102,103]^、N-糖链^[104,105]^、O-糖链^[103]^、糖胺聚糖^[106,107]^等在PGC上都能被很好地分离。因此,PGC-MS联用作为一种结构糖组学分析的常规方法被广泛应用于临床检测和新方法的开发。PGC-MS方法在一次分析中可以提供3组相对独立的信息,用于定性和半定量描述聚糖结构:聚糖组成和类型(MS信息)、聚糖的结构(色谱的保留时间和二级质谱信息)和聚糖的相对量(信号强度)。因此,针对临床组织病理学切片等可用材料非常有限的样品分析,PGC-MS具有明显的优势。Stadlmann等^[108]^采用PGC-MS方法高通量地比较分析了多克隆抗体和几种单克隆抗体IgG的糖组,发现PGC-MS可以在短时间内提供其他方法无法比拟的结果。Packer课题组利用PGC-MS糖组学方法研究了不同癌症细胞系(如结直肠癌^[109]^、白血病^[110]^等)中蛋白糖基化的变化;Stavenhagen等^[111]^详尽地综述了PGC-MS在临床上的应用;Abrahams等^[112]^利用先前报道的从IgG、免疫球蛋白A(IgA)、乳铁蛋白、核糖核酸酶B、胎球蛋白和卵清蛋白释放的N-糖结构,总结了它们在PGC-MS上的行为,创建了一个N-聚糖PGC保留文库(http://www.glycostore.org/showPgc),为科学家们提供了全面的参考。此外,在基于PGC-MS的新方法开发方面,Li等^[113]^发展了纳升级PGC-LC-MS全面表征细胞糖萼中N-糖组、O-糖组和糖脂组方法,其中N-糖组和O-糖组分离的核心采用了PGC nanochip,该方法分析了脑细胞糖萼中糖链的组成,检测到超过600种N-糖链和超过70种O-糖链。Hinneburg等^[114]^提出了一种基于PGC-MS的毛细管流配置方法,在PGC分离后、MS检测前,利用T型管路配件向分离后的聚糖泵入离子促进剂,显著提高了糖链检测的灵敏度、覆盖率和定量准确性,为难以检测的低丰度聚糖分析提供了新的解决方案;Zhang等^[115]^建立了一个基于聚偏二氟乙烯(PVDF)膜固定化的细胞N-糖组和O-糖组分析方法,释放的聚糖可以在96孔板中被还原、脱盐、纯化和重组,无需额外染色或衍生;该方法采用了纳升级PGC色谱柱(100 mm×75 μm)对糖链进行分离,对小鼠乳腺细胞释放的N-糖组和O-糖组分离结果如图4所示。

**图4:**
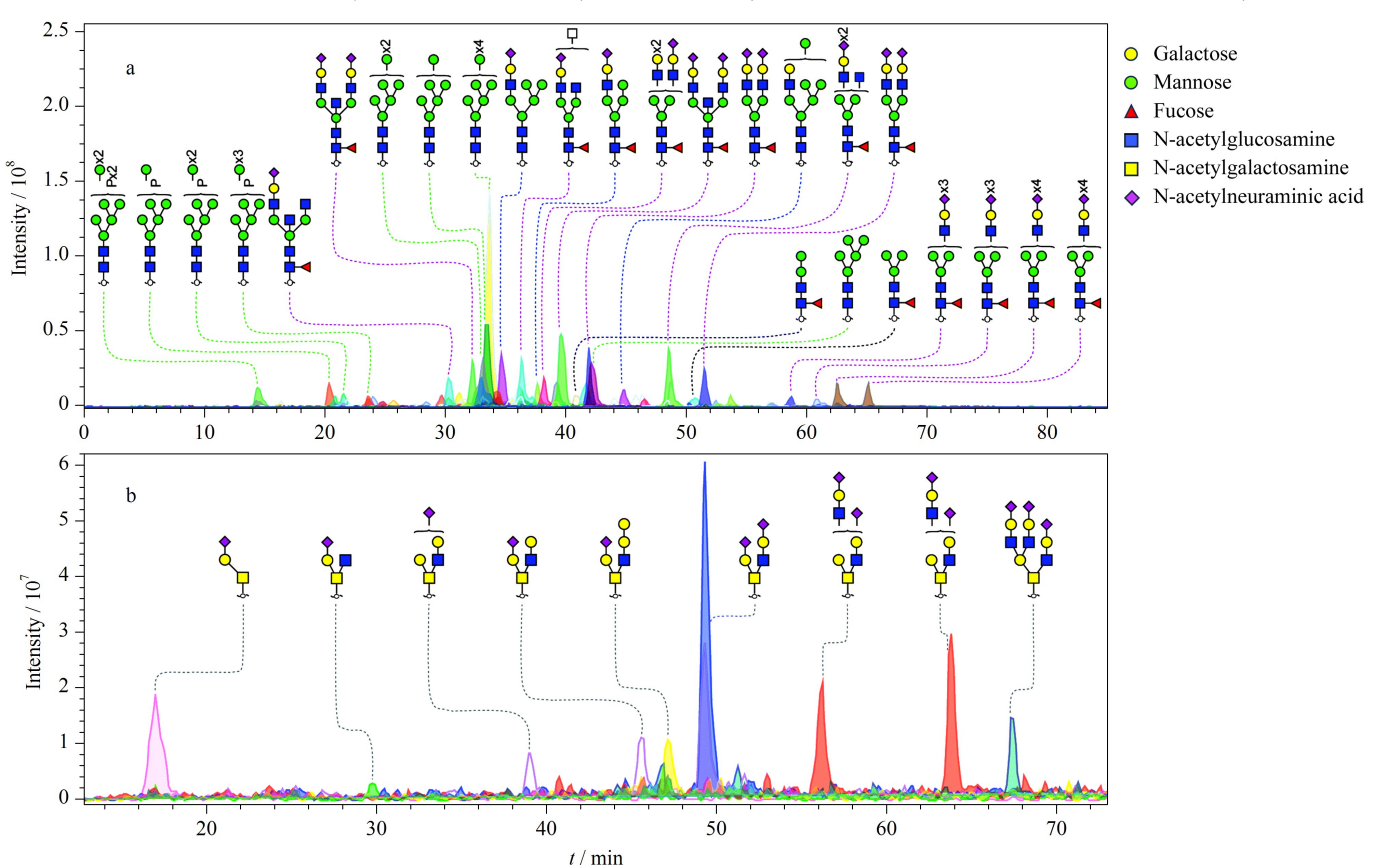
小鼠乳腺细胞N-糖组和O-糖组的PGC nano-LC-ESI-MS/MS分析

### 2.5 各类液相色谱技术的比较

在糖组学分析中,各类色谱模式都有成功的应用案例。但其中,应用最为广泛的是HILIC和PGC色谱。HPAEC因仪器配置(在线脱盐抑制器)的限制应用较少,RP-HPLC也因其对糖链部分的分离贡献较小而鲜被报道。HILIC模式常被用于衍生化糖链的分离,最为常见的是对2-AB衍生化糖链的分离。糖链被衍生后电离效率和可检测性显著提高,低丰度的糖链更易被检测,但衍生化操作过程相对繁琐,副反应或副产物会不可避免地影响检测结果的准确性和完整性^[82]^。PGC色谱对非衍生的糖链分离效果好,但受糖链自身性质的影响,低丰度糖链的检测完整性仍存在问题。针对特定样品或目标分析物选择合适的分析方法,或采用哪种方法相互验证是值得研究者思考的问题。此外,由于聚糖含量差异较大且结构复杂,异构体数目较多,单一的色谱模式很难实现复杂糖链混合物的分离。为了获取最佳的正交性、峰容量和分离选择性,多维色谱技术是解决此类问题的策略之一^[18,32,94,116⇓-118]^。例如,Song等^[18]^采用三维色谱(RP-HPLC×HILIC×PGC)方法实现了毫克级2-氨基-*N*-(2-氨基乙基)-苯甲酰胺(AEAB)标记的鸡卵清蛋白N-糖链的纯化制备;Alley等^[118]^采用循环HILIC(Amide-80)多维色谱模式,实现了4-AB标记的N-糖链纯化制备,并将其应用于芯片制备;对于质谱响应高的唾液酸化糖链和中性糖链混合物的分离,可以采用第一维色谱将两类糖分离,第二维色谱对其分别进行分离分析,可有效减少唾液酸化糖对中性糖的电离抑制。对于含量差异较大的糖链分离,亦可采用多维色谱的模式,将高含量和低含量糖链分别分析,保证糖链结构鉴定的完整性等。

## 3 结论与展望

糖组学研究对了解生命活动规律以及疾病的预防和治疗具有重要意义。但由于糖链在生物样品中的丰度低、含量差异大、在质谱中响应较低等因素,糖链的精细结构解析仍存在巨大挑战。液相色谱技术参与了糖组学分析策略中的糖链富集和分离等关键过程,对提高糖链的丰度与纯度,糖链结构的准确解析发挥了不可或缺的作用。此外,液相色谱技术对于寡糖的定量分析也至关重要。良好的色谱分离技术,不仅能够提高可定量寡糖的种类与数目,也是糖链定量结果准确的重要保障。目前的研究报道中,糖链或糖肽的分离应用最为广泛的是HILIC和PGC色谱,但针对特定样品或目标分析物选择合适的分析方法是值得研究者思考的问题。近年来,微升级、纳升级的超高效液相色谱系统被广泛应用于天然化合物的分离分析,其优秀的分离能力和低样品消耗符合糖组学的分析要求,是未来糖组学色谱分析的发展方向。如何提高色谱材料对不同类型糖分子的选择性仍是色谱材料开发的关键问题。此外,糖组学研究尚缺乏统一的标准,操作的规范化有待提高,检测的准确性有待科学地评估。尽管近年来糖组学研究取得了相当大的进展,但研究人员仍然面临着重大挑战。相信随着新材料和色谱技术的不断发展,色谱分离技术在糖组学研究中将发挥更重要的作用。
